# TMP195 Exerts Antitumor Effects on Colorectal Cancer by Promoting M1 Macrophages Polarization

**DOI:** 10.7150/ijbs.73264

**Published:** 2022-09-06

**Authors:** Yicheng Han, Jiachun Sun, Yanyan Yang, Yunlong Liu, Jun Lou, Hongming Pan, Junlin Yao, Weidong Han

**Affiliations:** 1 Department of Medical Oncology, Sir Run Run Shaw Hospital, College of Medicine, Zhejiang University, Hangzhou, Zhejiang, China.; 2 The First Affiliated Hospital, and College of Clinical Medicine of Henan University of Science and Technology, Luoyang 471003, China.

**Keywords:** Colorectal cancer, TMP195, HDAC, macrophage, PD-1 blockade

## Abstract

Studies have shown that epigenetic enzymes such as histone deacetylase (HDAC) are closely related to cancers and that several HDAC inhibitors exert antitumor effects. Studies have further suggested that class IIa HDAC inhibitors are related to immune functions, including immune responses and the expression of chemokines and complement pathway components. TMP195, a selective class IIa HDAC inhibitor, has been reported to be effective against breast cancer. However, the role and mechanism of TMP195 in colorectal cancer remain unknown. In this study, we found that TMP195 significantly reduced the tumor burden in two mouse models of colitis-associated colorectal cancer (CAC) and subcutaneous tumor. Mechanistically, TMP195 decreased the proportion of total macrophages but increased the proportion of M1 macrophages by promoting polarization, resulting in the increased release of inflammatory cytokines. TMP195 had no direct effect on the proliferation of colorectal cancer cells, and its antitumor effect on the colorectal cancer disappeared when macrophages were partly depleted by clodronate liposomes. In addition, TMP195 enhanced the efficacy of PD-1 blockade. The present study revealed that the combination of TMP195 and PD-1 blockade may provide a therapeutic strategy for colorectal cancer.

## Introduction

Colorectal cancer (CRC) is the third most common malignant cancer and the second leading cause of cancer-related death worldwide, but there is still a lack of effective understanding of its initiation and development mechanisms 1. The five-year survival rate of patients diagnosed with metastatic colorectal cancer is less than 20% 2, 3. At present, in addition to classic chemotherapy, targeted therapy and immunotherapy have gradually become important cornerstones of colorectal therapy 4. Recent studies have reported that targeted therapies, such as bevacizumab, which is a monoclonal antibody against vascular endothelial growth factor (VEGF), combined with chemotherapy can improve the survival rates of patients with metastatic CRC 5. Studies on immunotherapy have reported that immune checkpoint inhibitors (ICIs) have significant therapeutic effects on specific CRC subtypes, including mismatch repair-deficient (dMMR) and microsatellite instability high (MSI-H) CRC 4, 6. During the treatment of CRC with ICIs, in addition to T cells, innate immune cells, such as macrophages, dendritic cells and natural killer cells, also infiltrate the microenvironment of dMMR and MSI-H CRC tumors 7, 8. In the microenvironment of CRC tumors, a high proportion of tumor-associated macrophages (TAMs) express programmed cell death protein 1 (PD-1), which is negatively correlated with phagocytic effects against tumor cells 9. The use of checkpoint inhibitors targeting PD-1 or programmed cell death protein 1 ligand 1 (PD-L1) increases macrophage phagocytosis and suppresses tumor growth 9. Therefore, macrophage modulators are currently worth studying for treating CRC tumors 10. In the past decade, new drugs have roughly doubled the average survival time of patients with advanced colorectal cancer, but patients usually die within three years 11. Therefore, it is critical to identify reliable new drugs for the treatment of colorectal cancer.

Previous studies have shown that the expression of epigenetic enzymes is often dysregulated in human tumors, which is closely related to the occurrence and development of tumors 12. Histone deacetylase (HDAC) is a critical epigenetic enzyme that regulates the deacetylation of genes and affects expression by removing acetyl groups from histones 13, 14. HDAC inhibitors (HDACis) can inhibit HDAC-mediated deacetylation, leading to histone hyperacetylation and the re-expression of epigenetically silenced genes 15. Based on their homology to yeast proteins, HDACs are classified as class I (HDACs 1, 2, 3 and 8), class IIa (HDAC4, -5, -7 and -9), class IIb (HDAC6 and -10), class III (SIRTs 1-7)s and class IV (HDAC11) 16. Accordingly, HDAC inhibitors can be divided into class I HDACis and class II HDACis. HDACis with different specific characteristics have different antitumor mechanisms. HDACis exert antitumor effects by regulating tumor cell apoptosis, growth arrest, senescence, differentiation, and immunogenicity and inhibiting tumor angiogenesis 13, 17. The most representative HDACis are vorinostat and romidepsin, which have been approved by the FDA and successfully used in the clinical treatment of refractory cutaneous and peripheral T-cell lymphoma 18, 19. Compared with that affected by class I HDAC inhibitors such as vorinostat, gene expression affected by class IIa HDAC inhibitors is primarily related to immune function, including immune responses and the expression of chemokines and complement pathway components 20. TMP195 is a selective class IIa HDAC (HDAC4, -5, -7, and -9) inhibitor that has been reported to reprogram tumor-associated macrophages into tumoricidal cells and reduce tumor burden in breast cancer 21. The role and mechanism of TMP195 in other types of cancer have not been reported.

Here, we examined the therapeutic effect of TMP195 on colorectal cancer and its mechanism. The results demonstrated that the class IIa HDAC inhibitor TMP195 could reduce tumor burden by promoting M1 macrophage polarization, which exerted antitumoral effects on colorectal cancer. In addition, TMP195 could enhance the effect of immunotherapy (PD-1 blockade) on colorectal cancer.

## Materials and Methods

### Mice

C57BL/6 mice (male, 6-8 weeks old, 20-25 g, Shanghai Institute of Material Medicine, Chinese Academy of Science, China) were raised in the pathogen-free facility and all experiments were carried out in accordance with protocols approved by the Animal Care and Use Committee of Zhejiang University. All mice were euthanized before reaching a humane endpoint, with tumor growth no greater than 2 cm, or a total mass >10% of the mouse's weight.

### Mouse models

#### Colitis-associated colorectal cancer model

Colitis-associated colorectal cancer (CAC) was induced in 6-8 weeks old male C57BL/6 mice according to a previously reported method 22. The mice were intraperitoneally injected with 10 mg/kg azoxymethane (AOM, MP Biomedicals, USA) and then administered a normal diet and water for 7 days. Next, the mice were administered DSS (2%, w/v, MP Biomedicals) instead of water for 7 days, and during the recovery stage, the mice were given water (14 days). This process was repeated three times. Between the 85^th^ day and the 113^th^ day, mice that developed advanced cancer were given water and treated intraperitoneally with 50 mg/kg TMP195 dissolved in dimethyl sulfoxide (DMSO) or an equal volume of DMSO daily in the control group. Then, the mice were sacrificed, and peripheral blood and colon tissue were collected for subsequent analysis.

#### Subcutaneous graft tumor model

For the transplanted tumor model, 6-8-weeks-old male C57BL/6 mice were engrafted with MC38 colon carcinoma cells in the back of the right hindlimb by subcutaneously injecting 1×10^6^ cells in a 100 μL suspension. On the fifth day after MC38 cells were inoculated, the tumor-bearing mice were randomly divided and treated intraperitoneally with 50 mg/kg/day TMP195 dissolved in DMSO or an equal volume of DMSO as the control group. Approximately 20 days after subcutaneous inoculation, the mice were sacrificed, and peripheral blood and tumors were harvested. The size and weight of the tumors were measured, and the tumor tissue was used for flow cytometry and Q-PCR.

### Bone marrow-derived macrophage isolation

Six- to eight-week-old male C57BL/6 mice were sacrificed, and the tibia and femur were isolated under aseptic conditions. The muscle tissue was removed from the bone, and then both ends of the tibia and femur were removed with scissors. The bone was placed in a sterile PBS environment. A syringe filled with PBS was inserted into the bone marrow cavity to flush out the bone marrow contents. Bone marrow cells were centrifuged at 1500 rpm for 5 minutes and resuspended in red blood cell lysis to lyse red blood cells. After being filtered and centrifuged, the cells were resuspended in Dulbecco's modified Eagle medium (DMEM) (Geneodx, Shanghai, China) containing 10% fetal bovine serum (FBS, Gibco) and 100 ng/mL M-CSF (Peprotech, USA) and then seeded in cell culture dishes.

### Flow cytometry

Tumors were minced and digested with collagenase IV in a 37 ℃ shaker for 2 hours. After being filtered, the suspensions were centrifuged at 4 ℃ and 400 × g for 10 minutes and then resuspended in PBS. The cells in the suspensions were labeled alive or dead with a dead cell stain kit for 30 minutes (FITC LIVE/DEAD® Fixable Green Dead Cell Stain Kit, Thermo Fisher cat. L34970). After further centrifugation, the cells were resuspended in FACS buffer (PBS+2% FBS) to make a single-cell suspension. Before being stained, the samples were incubated with anti-mouse CD16/CD32 Fcγ receptor II/III blocking antibody (BioLegend cat. 101301, USA) for 15 minutes on ice in the dark environment. The cells were washed twice and incubated with fluorochrome-conjugated antibodies (APC anti-mouse CD45, clone 30-F11, BioLegend cat. 103112; Pacific Blue™ anti-mouse CD45, clone 30-F11, BioLegend cat. 103126; PerCP/Cyanine5.5 anti-mouse CD3ε, clone 145-2C11, BioLegend cat.100328; Brilliant Violet 605™ anti-mouse CD4, RM4-5, BioLegend cat. 100548; PE anti-mouse CD8a, clone 53-6.7, BioLegend cat. 100708; PerCP/Cyanine5.5 anti-mouse/human CD11b, clone M1/70, BioLegend cat. 101228; Brilliant Violet 421™ anti-mouse/human CD11b, clone M1/70, BioLegend cat. 101235; Brilliant Violet 650™ anti-mouse F4/80, clone BM8, BioLegend cat. 123149; PE anti-mouse F4/80, clone BM8, BioLegend cat. 123110; APC/Cyanine7 anti-mouse MHC II, clone M5/114.15.2, BioLegend cat. 107628; PE anti-mouse Ly-6G/Ly-6C (Gr-1), clone RB6-8C5, BioLegend cat. 108407) for 30 minutes at 4 ℃ in the dark. The single-cell suspensions prepared from tumor tissue were used to examine the tumor immune microenvironment, including T cells, macrophages, and myeloid-derived suppressor cells (MDSCs) by flow cytometry.

Macrophage: CD45^+^, CD11b^+^, F4/80^+^.

M1 macrophage: CD45^+^, CD11b^+^, F4/80^+^, MHC-II^+^.

Helper T cell: CD45^+^, CD3^+^, CD4^+^.

Cytotoxic T cell: CD45^+^, CD3^+^, CD8^+^.

MDSC: CD45^+^, CD11b^+^, Gr-1^+^.

### Cell culture

The HCT116, LoVo, and MC38 cell lines were purchased from the Cell Bank of the Chinese Academy of Science (Shanghai, China). HCT116 and MC38 cells were cultured in DMEM containing 10% inactivated FBS, 100 U/mL penicillin, and 100 μg/mL streptomycin at 37 °C in 5% CO_2_. LoVo cells were cultured in RPMI 1640 medium containing 10% FBS, 100 U/mL penicillin and 100 μg/mL streptomycin.

### RNA extraction and quantitative real-time polymerase chain reaction

Total RNA was extracted from cells or tissues by using RNAiso Plus reagent (#9109, Takara, Tokyo, Japan) in accordance with the instructions. The RNA was reverse-transcribed to cDNA using a HiScript II 1st Strand cDNA Synthesis Kit (#R312-02, Vazyme). Quantitative real-time PCR was performed using MagicSYBR Mixture (#CW3008M, CWbiotech, Beijing, China) according to the manufacturer's protocols. Target gene expression was standardized to β-actin gene expression. The primer sequences are listed in Table 1.

### Cytokine assay

The levels of cytokines interleukin-1β (IL-1β), IL-6, IL-12, and tumor necrosis factor-α (TNF-α) in the supernatant of cultured BMDMs and the serum of experimental mice were analyzed by using enzyme-linked immunosorbent assay (ELISA) kits (#88-7324-22, #88-7013-22, #88-7064-22, #88-7120-22, Invitrogen, CA, USA). The procedures were performed strictly according to the reagent instructions. The results were reported as pg/mL.

### Western blotting

Proteins were extracted with radioimmunoprecipitation assay (RIPA) buffer containing protease inhibitor cocktails (#C14012, Biotool, Beijing, China) and phosphatase inhibitors (#B15001-A&B, Biomake, Shanghai, China). The proteins were separated by size by SDS-polyacrylamide gel electrophoresis and transferred to polyvinylidene fluoride (PVDF) membranes (Bio-Rad, CA, USA). The membranes were incubated with the primary antibodies listed in Table 2 at 4 ˚C overnight. Then the membranes were incubated with peroxidase‑coupled secondary antibodies (CST, USA) at 25 ˚C for 2 hours. Signals were visualized by using an enhanced chemiluminescence kit (Amersham Imager 600, GE, MA, USA). Integrated density was analyzed by image J.

### Histopathology and immunostaining

The colon tissues of CAC mice and transplanted tumor tissues were fixed in 10% neutral-buffered formalin overnight at 4 °C. After being embedded in paraffin, the samples were prepared for hematoxylin and eosin (H&E) staining, immunohistochemistry (IHC) and immunofluorescence (IF) analysis. For IHC, tumor sections were stained with F4/80, CD8, PD-1 and PD-L1. The antibodies used for immunohistochemical staining are listed in table 2. After immunohistochemical staining, the samples were viewed and photographed under a positive fluorescence microscope (Leica DM4000). For IF staining, the samples were visualized by using 4´,6-diamidino-2-phenylindole (DAPI; #MA0128, Meilunbio) as a chromogen. Tumor tissues were examined with Alexa Fluor 488 (F4/80) and Alexa Fluor 594 (CD86).

### Statistical analysis

The data are presented as the mean ± standard error of the mean (SEM). Differences between groups were compared by using Student's t test and one-way ANOVA. All statistical analyses and graphs were generated using Prism (GraphPad Software, CA, USA). P values are designated as * P < 0.05, * * P < 0.01, * * * P < 0.001, and * * * * P < 0.0001, and P values < 0.05 indicated statistical significance.

## Results

### TMP195 has a significant therapeutic effect on CAC

**Figure 1:**
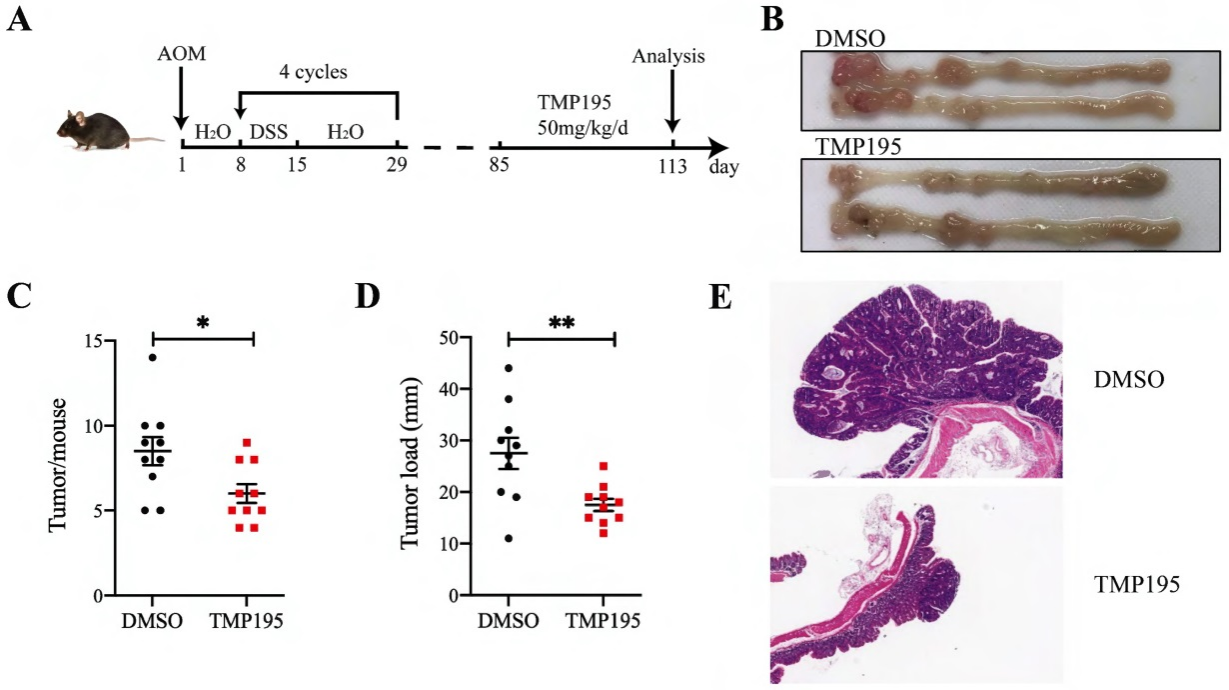
** TMP195 has a significant therapeutic effect on CAC. (A)** Schematic of the CAC model induced by AOM/DSS. Mice that developed advanced cancer were randomly placed into treatment groups. The mice were treated with DMSO or 50 mg/kg/d TMP195 from the 85^th^ day to the 113^th^ day. **(B)** Representative photographs of colons are shown.** (C)** The number of tumors and **(D)** tumor load per mouse were measured. **(E)** Colon tumor tissue was embedded in paraffin and stained with H&E. These two photographs were taken at the same magnification. (n = 10 per group) (P < 0.05 is statistically significant, *P < 0.05, **P < 0.01, ***P < 0.001)

As described in the methods, the CAC model was constructed by AOM/DSS administration, and the mice were treated with TMP195 during the advanced stage of colorectal cancer to study its therapeutic effect (Figure 1A). The mice were randomly divided into two groups (TMP195 group and DMSO solvent group as control group) on the 85^th^ day and injected with TMP195 (50 mg/kg/day, i.p., Selleck) or an equal volume of DMSO solvent for 28 days (the 85^th^ - 113^th^ day). The mice were sacrificed on the 113^th^ day, and the numbers and sizes of colon tumors were counted and recorded (Figure 1B). Compared with that in the control group, the number of tumors in the TMP195-treated group was significantly decreased (8.50 ± 0.83 vs. 6.00 ± 0.56, P < 0.05, Figure 1C). In addition, the tumor load in mice, which refers to the sum of the diameters of all tumors in each mouse, decreased significantly after TMP195 treatment (27.50 ± 3.02 vs. 17.50 ± 1.20 mm, P < 0.01, Figure 1D). Distal colons were embedded, and hematoxylin and eosin (H&E) staining was performed to evaluate tumor histomorphology. Histologically, two representative colon sections from each group are shown, and less aggressive adenocarcinomas and smaller tumors were observed in the TMP195-treated group (Figure 1E).

### TMP195 has a therapeutic effect on MC38-transplanted tumors in C57BL/6 mice

**Figure 2:**
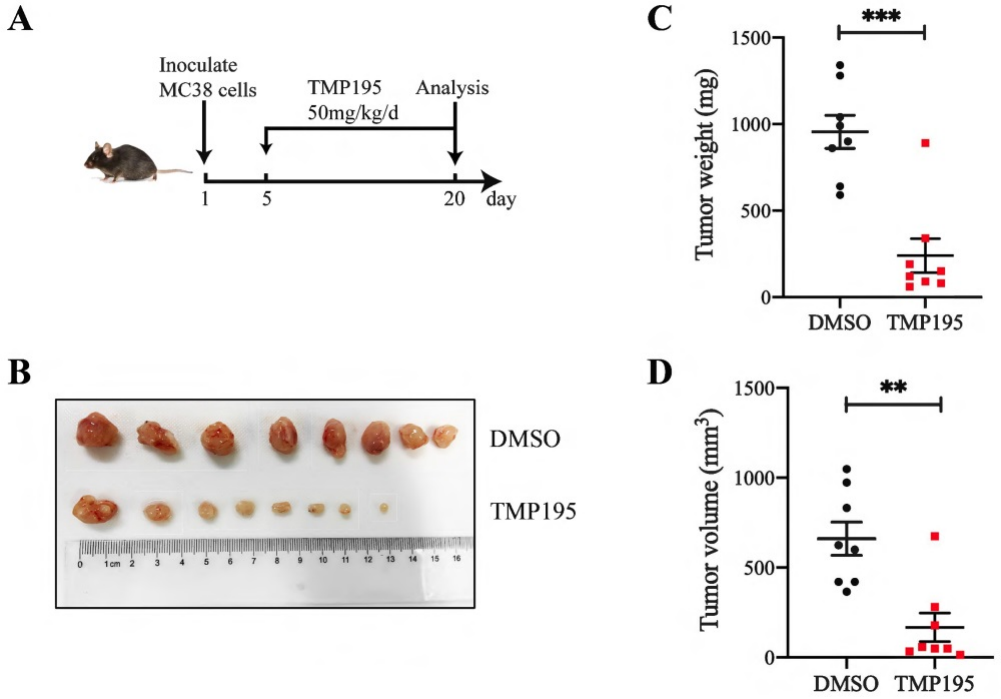
** TMP195 has a therapeutic effect on MC38-transplanted tumors in C57BL/6 mice. (A)** Schematic showing the subcutaneously transplanted tumor model established in C57BL/6 mice with the murine colon cancer cell line MC38. On the 5^th^ day, tumor-bearing mice with similar tumor volumes were randomly divided into treatment groups and administered 50 mg/kg/d TMP195 or DMSO until Day 21. **(B)** The tumors were harvested and photographed. **(C)** Tumor weight and **(D)** volume were measured in each group. (n = 8 per group) (*P* < 0.05 is statistically significant, *P < 0.05, **P < 0.01, ***P < 0.001)

To further confirm the therapeutic effect of TMP195 on colorectal cancer in mice, a subcutaneously transplanted tumor model was established in C57BL/6 mice with murine colon cancer cell line MC38. The mice were administered 50 mg/kg/day TMP195 by intraperitoneal injection between the 5^th^ day and 20^th^ day (Figure 2A). The mice were euthanized, and the tumors in the back of the right hindlimb were harvested. Tumor size and weight were measured (Figure 2B). Compared with those in the control group, the weight (955.0 ± 95.13 vs. 240.0 ± 98.02 mg, P < 0.001, Figure 2C) and volume (660.7 ± 92.97 vs. 167.2 ± 79.12 mm^3^, P < 0.01, Figure 2D) of the transplanted tumors in the TMP195-treated group were significantly lower.

### TMP195 has no direct effect on colorectal cancer cell proliferation or apoptosis *in vitro*

To investigate the mechanism of the therapeutic effect of TMP195 on colorectal cancer, we examined whether TMP195 had a direct effect on colorectal cancer. MC38 cells were cultured in DMEM containing 5 μM, 20 μM, 40 μM or 60 μM TMP195, and there was no significant difference in MC38 cell proliferation or apoptosis between different groups (Supplemental Data Figure 1A-B). In the HCT116 and LoVo human colorectal cancer cells, TMP195 did not directly promote or inhibit cell proliferation or apoptosis (Supplemental Data Figure 1C-F).

Most cells are more resistant to chemotherapeutics when they grow on hard plastic surfaces compared to soft matrices *in vitro*. To further clarify whether TMP195 has no direct effect on colorectal cancer cell proliferation and apoptosis, a soft agar colony formation assay was performed, and the cells were grown independently of a solid surface. MC38 cells were cultured in soft agar, with different concentrations of TMP195 (0 μM, 5 μM, 20 μM, 40 μM and 60 μM). There was no significant difference in proliferation rate among these groups. (Supplemental Data Figure 1G-H).

### TMP195 decreases the macrophage-to-leukocyte proportion in CAC tumors and the surrounding lamina propria

**Figure 3:**
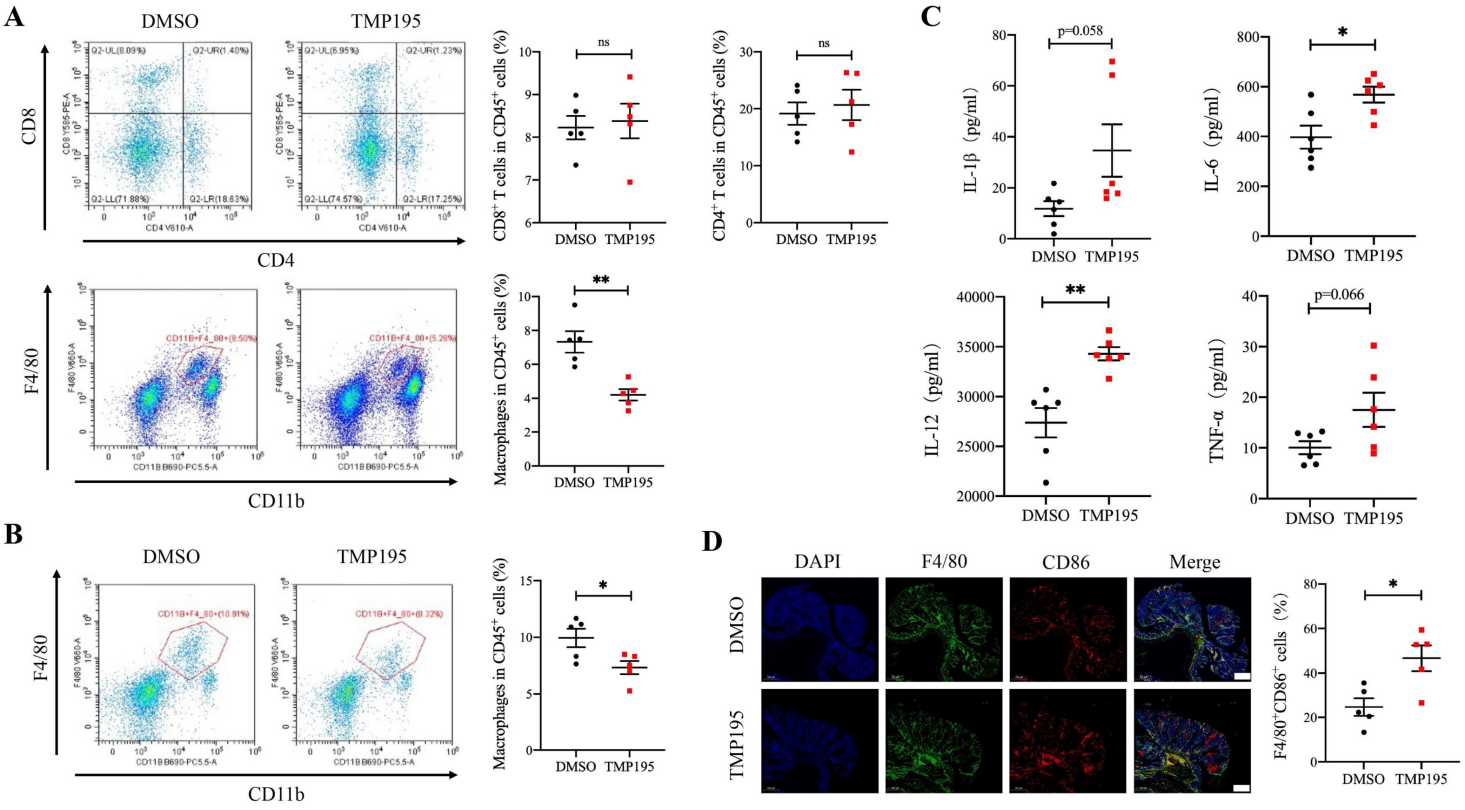
** TMP195 decreases the macrophage-to-leukocyte proportion in CAC tumors and the surrounding lamina propria.** CAC mice were treated with TMP195 or DMSO and sacrificed on Day 113. Tumor tissue and lamina propria tissue in the colon were extracted and digested into single-cell suspensions with collagenase IV.** (A)** Macrophage and T lymphocyte infiltration in the tumor tissue of CAC mice was examined by flow cytometry. The immune cell infiltration in tumor tissue were plotted as a percentage of CD45^+^ cells. Macrophages were identified with CD45^+^CD11b^+^F4/80^+^. CD45^+^CD8^+^ T cells and CD45^+^CD4^+^ T cells indicate cytotoxic T cells and helper T cells respectively. (n = 5 per group) **(B)** Macrophage infiltration in the lamina propria was detected by flow cytometry and plotted as a percentage of CD45^+^ cells. (n = 5 per group) **(C)** The concentrations of the cytokines IL-1β, IL-6, IL-12 and TNFα in the peripheral blood of CAC mice treated with 50 mg/kg/d TMP195 or DMSO were examined by ELISA. (n = 6) **(D)** The tumor sections from CAC mice treated with 50 mg/kg/d TMP195 or DMSO were used for IF staining. Proinflammatory macrophage was identified by the colocalization of F4/80 and CD86, and plotted as a percentage of F4/80^+^ cells. Scale bars: 500 μm. (n = 5) (P < 0.05 is statistically significant, *P < 0.05, **P < 0.01, ***P < 0.001)

To further examine the mechanism of the therapeutic effect of TMP195, the infiltration of immune cells, including macrophages, cytotoxic T cells and helper T cells, in CAC tumor tissue was analyzed. These cells were reported to be closely related with CAC tumor progression. Mice with advanced CAC were treated with 50 mg/kg/d TMP195 or DMSO. Tumor tissue and lamina propria tissue of the colon were extracted and digested into single-cell suspensions with collagenase IV. Single-cell suspensions were prepared and examined by flow cytometry with fluorescein-labeled antibodies. The marker CD11b and F4/80 were used to identify macrophages. The marker CD4 and CD8 labeled helper T cells and cytotoxic T cells respectively. The results showed that the proportion of CD45^+^CD11b^+^F4/80^+^ macrophages relative to CD45^+^ leukocytes in the TMP195-treated group was significantly lower than that in the control group in tumor tissue, but there was no significant difference in the proportion of infiltrating cytotoxic T cells and helper T cells (Figure 3A). In the lamina propria of colon from CAC mice treated with TMP195, a decrease in the infiltration of total macrophages was also detected (Figure 3B). Previous studies reported that in AOM/DSS-induced CAC, macrophage depletion reduced tumorigenesis 23, 24. These results indicated that the therapeutic effect of TMP195 on CAC might occur via the regulation of macrophages. In addition, an increase in the level of inflammatory cytokines IL-1β, IL-6, IL-12 and TNFα was detected in the peripheral blood of CAC mice with TMP195 treatment (Figure 3C). This result indicated that TMP195 might exert a proinflammatory function. In order to further understand whether TMP195 has a proinflammatory effect in CAC tumor tissue, immunofluorescence was performed to analyze major proinflammatory cells. The proportion of F4/80^+^CD86^+^ proinflammatory macrophages relative to total macrophages in CAC was higher after TMP195 treatment (24.67 ± 4.01 vs. 46.69 ± 5.76%, P< 0.05, Figure 3D). These results suggested that TMP195 might play a therapeutic role by regulating macrophages, especially proinflammatory macrophages.

### TMP195 increases the proportion of M1 macrophages among total macrophages and promotes M1 macrophage-associated cytokine secretion in mice with MC38-transplanted tumors

**Figure 4:**
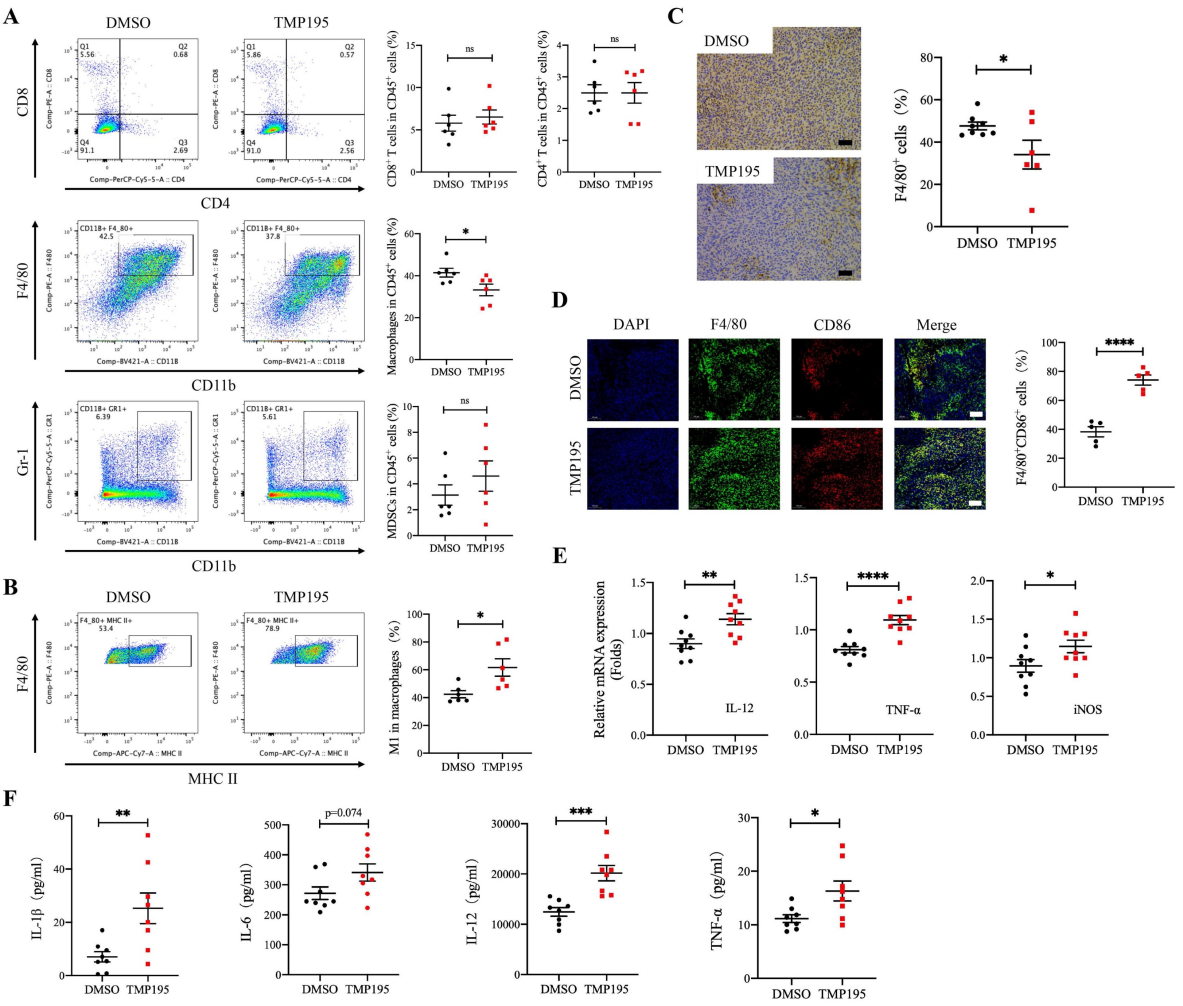
**TMP195 increases the proportion of M1 macrophages among total macrophages and promotes M1 macrophage-associated cytokine secretion in mice with MC38-transplanted tumors.** Tumors from the MC38-transplanted tumor mice were digested with collagenase IV, and immune cell infiltration in the tumors was examined by flow cytometry. **(A)** The proportion of CD45^+^CD11b^+^F4/80^+^ macrophages, CD45^+^CD8^+^ T lymphocytes, CD45^+^CD4^+^ T lymphocytes and CD45^+^CD11b^+^Gr-1^+^ MDSCs relative to CD45^+^ leukocytes in transplanted tumor tissue were analyzed. (n = 6 per group) **(B)** The proportion of CD45^+^CD11b^+^F4/80^+^MHC-II^+^ M1 macrophages relative to total macrophages in these samples was detected. (n = 6 per group) **(C)** IHC was performed on the transplanted tumor sections to examine the macrophage-specific marker F4/80. Representative photographs at the same magnification are shown. Scale bars: 50 μm. (n = 8 for the control group, n = 6 for the TMP195-treated group) **(D)** Immunofluorescence was performed to identify M1 macrophages expressing CD86 and F4/80, and the colocalization of these two markers was analyzed. The proportion of F4/80^+^CD86^+^ M1 macrophages was plotted as a percentage of F4/80^+^ cells. Representative photographs are showed. Scale bars: 100 μm. (n = 5 each group) **(E)** The MC38-transplanted tumor tissues were lysed to extract total RNA. The mRNA levels of the inflammatory cytokines IL-12, TNF-α and iNOS in MC38-transplanted tumor tissue were measured by qRT-PCR. (n = 9 per group) **(F)** The concentrations of the typical inflammatory cytokines IL-1β, IL-6, IL-12 and TNFα in the peripheral blood of mice with MC38-transplanted tumors were examined by ELISA. (n = 8 per group) (P < 0.05 is statistically significant, *P < 0.05, **P < 0.01, ***P < 0.001)

The immune microenvironment of MC38-transplanted tumor tissues was also analyzed by flow cytometry. Similar to the CAC mouse model, the results showed that the proportion of CD45^+^CD11b^+^F4/80^+^ macrophages relative to leukocytes was lower in the TMP195-treated group than in the control group (41.48 ± 2.08 vs. 33.25 ± 2.76%, P < 0.05, Figure 4A). There was no significant difference in the proportion of CD45^+^CD8^+^ cytotoxic T cells, CD45^+^CD4^+^ helper T cells or CD45^+^CD11b^+^Gr-1^+^ MDSCs between the TMP195 group and control group (Figure 4A). It is known that the main macrophages in tumor tissues are antitumor M1 macrophages and protumor M2 macrophages. In MC38-transplanted tumors, the proportion of CD45^+^CD11b^+^F4/80^+^MHC-II^+^ M1 macrophages relative to total macrophages was increased in the TMP195 treatment group compared with the control group (42.45 ± 2.56 vs. 61.72 ± 6.26%, P < 0.05, Figure 4B). Immunohistochemical staining was performed on the tumor tissue samples. The results showed that the positive rate of the macrophage marker F4/80 decreased after TMP195 treatment, and a representative sample is shown (47.64 ± 1.79 vs. 34.11 ± 6.79%, P < 0.05, Figure 4C). The proportion of F4/80^+^CD86^+^ M1 macrophage was also analyzed by immunofluorescence staining, and M1 macrophages showed markedly more infiltration in the TMP195-treated group than in the control group (38.26 ± 3.50 vs. 74.02 ± 3.48%, P < 0.0001, Figure 4D). *In vivo*, TMP195 increased the proportion of M1 macrophages relative to total macrophages.

In MC38-transplanted tumor tissue, TMP195 not only increased the proportion of M1 macrophages but also promoted the expression of M1 macrophage-associated inflammatory cytokines, including IL-12, TNFα and inducible nitric oxide synthase (iNOS) (Figure 4E). These results showed the RNA levels, and the protein levels were also examined. The peripheral blood was collected, and serum levels of the inflammatory cytokines IL-1β, IL-12 and TNFα in the TMP195 group were significantly higher than that in the control group (Figure 4F).

### Macrophage depletion can abrogate the therapeutic effect of TMP195 on colorectal cancer

**Figure 5:**
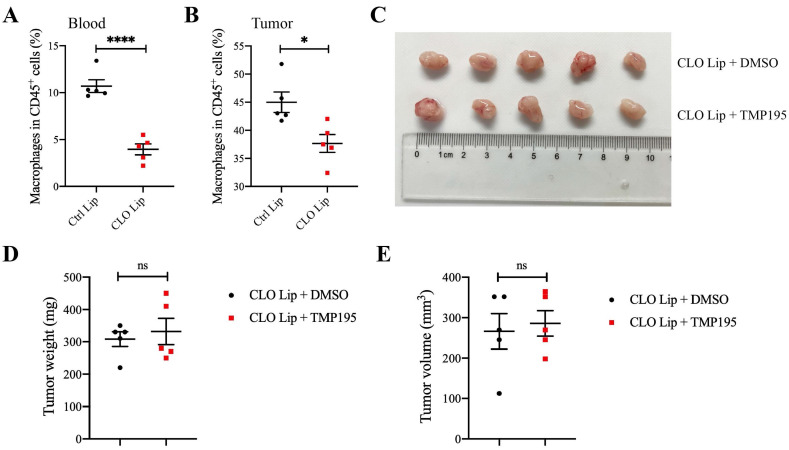
**Macrophage depletion can abrogate the therapeutic effect of TMP195 on colorectal cancer**. Clodronate liposomes can partly eliminate macrophages. The mice were divided randomly into two groups: clodronate liposomes (CLO Lip) and control liposomes (Ctrl Lip). Clodronate liposomes (CLO Lip) were administered intraperitoneally (200 μl) every three days beginning two days before MC38 inoculation until the mice were sacrificed, and 200 μl of control liposomes were administered to the control group. Macrophages in MC38-transplanted tumor tissues **(B)** and peripheral blood **(A)** were analyzed by flow cytometry. (n = 5 each group)** (C)** Representative photographs of tumors in the control group (CLO Lip plus DMSO group) and TMP195 group (CLO Lip plus TMP195 group) are shown. Mice in both groups were administered clodronate liposomes to eliminate macrophages. **(D)** Tumor weight and **(E)** volume were measured in each group. (n = 5 per group) (P < 0.05 is statistically significant, *P < 0.05, **P < 0.01, ***P < 0.001)

To verify whether macrophages play a decisive role in the therapeutic effect of TMP195 on CRC, mice with MC38-transplanted tumors were administered clodronate liposomes (CLO lip, Vrije University, Holland) to deplete some macrophages. Clodronate liposomes or control liposomes were intraperitoneally injected (200 µl) every three days beginning two days before MC38 inoculation until the mice were sacrificed. The proportion of peripheral blood macrophages was examined by flow cytometry, and approximately half of the macrophages were depleted (10.70 ± 0.68 vs. 3.95 ± 0.58%, P<0.001, Figure 5A). To verify that clodronate liposomes could reduce the number of macrophages in tumor tissue, mice that were grafted with MC38-transplanted tumors were divided into two groups: the CLO lip group and the control liposome (Ctrl Lip) group. The proportion of macrophages in tumor tissue was analyzed by flow cytometry. Clodronate liposomes could reduce the proportion of macrophages in tumor tissue (44.98 ± 1.83 vs. 37.66 ± 1.59%, P<0.05, Figure 5B).

Mice with MC38-transplanted tumors were all administered clodronate liposomes to deplete macrophages and then randomly divided into two groups: the DMSO control group and the TMP195 treatment group. TMP195 was administered in the same way as described above. After the mice were sacrificed, the tumors were photographed (Figure 5C). Tumor size and weight were measured and analyzed (Figure 5D-E). There was no significant difference in tumor size or weight between the two groups, which indicated that TMP195 lost its therapeutic effect on colorectal cancer after macrophage depletion.

### TMP195 promotes M1 macrophage polarization induced by LPS

**Figure 6:**
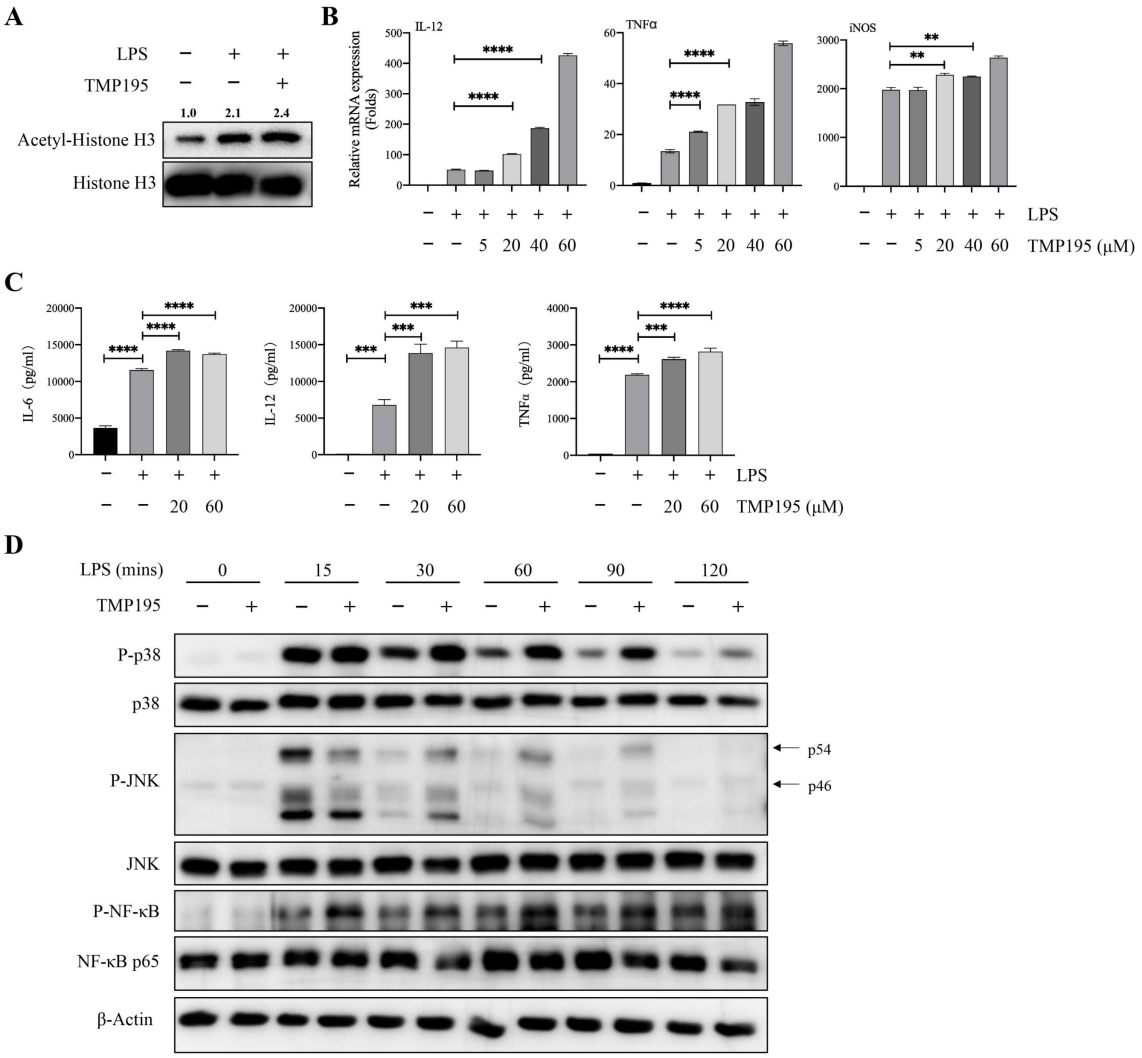
** TMP195 promotes M1 macrophage polarization induced by LPS. (A)** BMDMs were induced to undergo M1 polarization with 100 ng/ml LPS and 40 μM TMP195 for 2 hours, and proteins were extracted for western blotting with histone H3 and acetyl-histone H3 antibody. Relative integrated density (acetyl-histone H3 to histone H3) was calculated. **(B)** BMDMs were treated with LPS (100 ng/mL) and TMP195 (5, 20, 40 or 60 μM) for 4 hours. The mRNA levels of inflammatory cytokines IL-12, TNF-α and iNOS were analyzed by qRT-PCR. **(C)** BMDMs were administered 100 ng/ml LPS alone or in combination with 20 or 60 μM TMP195 for 8 hours, and the concentration of cytokines IL-6, IL-12 and TNFα in the supernatant was examined by ELISA. **(D)** BMDMs were stimulated with 100 ng/ml LPS and 40 μM TMP195 for different times (0, 15, 30, 60, 90, 120 mins). Proteins were extracted for immunoblotting with the indicated antibodies. (P < 0.05 is statistically significant, *P < 0.05, **P < 0.01, ***P < 0.001)

Bone marrow-derived macrophages (BMDMs) were used to further study whether TMP195 promoted the function of M1 macrophages. BMDMs were isolated and stimulated with the macrophage colony-stimulating factor (M-CSF) for five days. Five days after isolation, 100 ng/ml LPS alone or with 40 μM TMP195 was administered to induce BMDMs to differentiate into M1 macrophages for 2 hours. Total proteins were collected. To determine whether TMP195 works on BMDMs *in vitro* can be reflected by the level of histone acetylation. The protein levels of histone H3 and acetyl-histone H3 were examined by western blot. Integrated density of acetyl-histone H3 relative to histone H3 was analyzed. BMDMs stimulated with LPS and TMP195 exhibited higher level of histone acetylation (relative integrated density 2.4 vs. 2.1, Figure 6A). This result indicated that TMP195, as a HDAC inhibitor, led to histone hyperacetylation and influenced the re-expression of epigenetically silenced genes. To examine the expression of M1 macrophage-associated inflammatory cytokines, LPS and different concentrations of TMP195 (5 μM, 20 μM, 40 μM and 60 μM) were administered for 4 hours to extract total RNA. The mRNA expression of M1 macrophage-associated inflammatory cytokines, including IL-12, TNFα and iNOS, was measured (Figure 6B). The protein levels of inflammatory cytokines were examined by ELISA. BMDMs were stimulated with TMP195 (20 μM or 60 μM) for 8 hours, and the culture medium was collected. The expression of the typical inflammatory cytokines IL-6, IL-12 and TNFα in the TMP195 group was significantly higher than that in the LPS group (Figure 6C). To further study the mechanism by which TMP195 promotes inflammatory cytokine secretion, we evaluated whether TMP195 could regulate crucial inflammatory cytokine-associated signaling pathways in BMDMs by western blotting. TMP195 increased the phosphorylation of p38 MAPK, JNK and NF-κB p65 (Figure 6D). Based on these results, we hypothesized that TMP195 might promote M1 macrophage polarization and function via the MAPK and NF-κB signaling pathways.

### TMP195 may enhance the efficacy of PD-1 blockade

**Figure 7:**
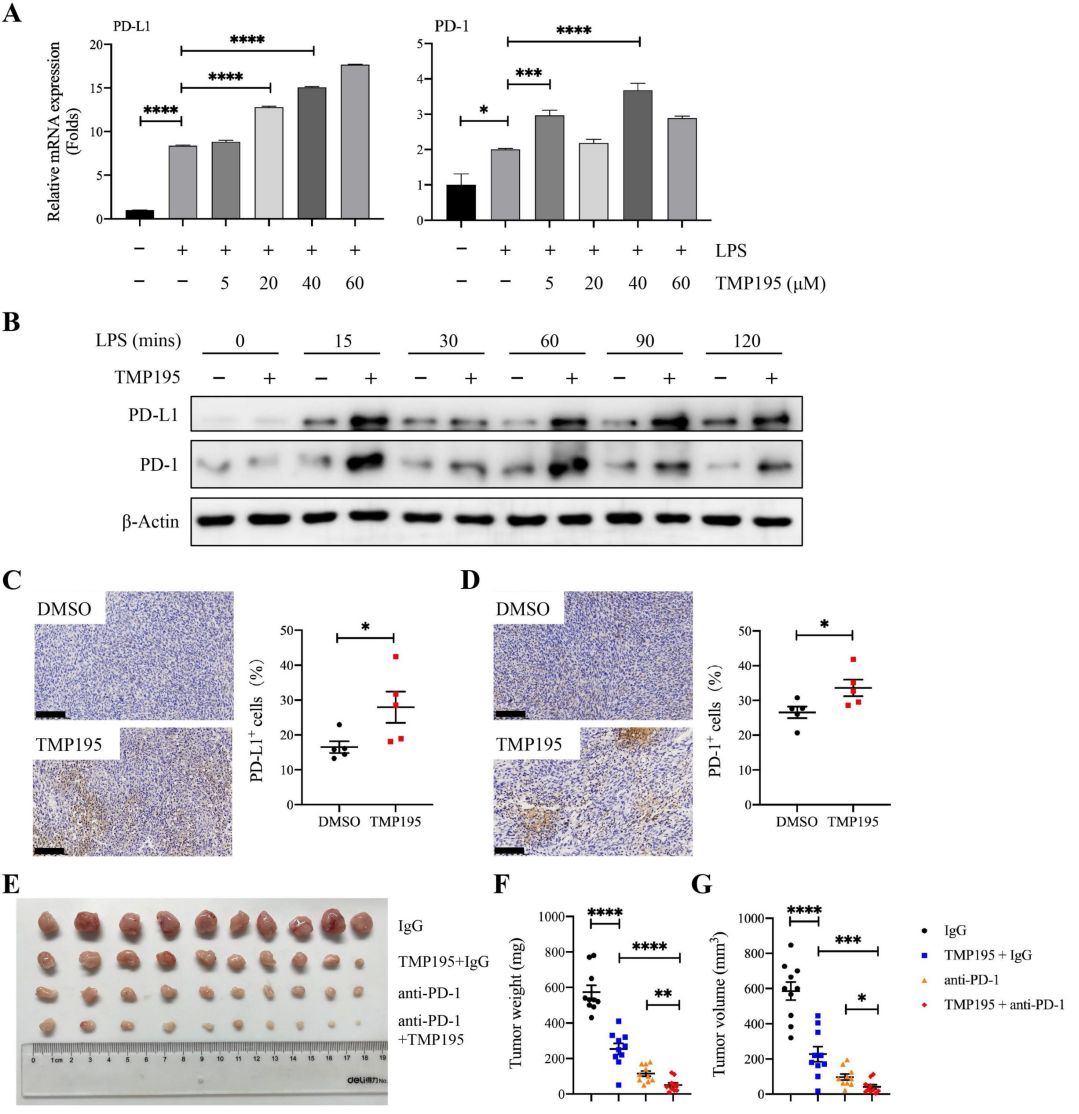
** TMP195 may enhance the efficacy of PD-1 blockade. (A)** BMDMs were treated with LPS (100 ng/mL) and TMP195 (5, 20, 40 or 60 μM) for 4 hours, and the mRNA levels of PD-1 and PD-L1 were measured by qRT-PCR. **(B)** BMDMs were stimulated with 100 ng/ml LPS and 40 μM TMP195 for different times (0, 15, 30, 60, 90, 120 mins). The protein levels of PD-1 and PD-L1 were examined by immunoblotting. **(C-D)** IHC was performed on the transplanted tumor sections to examine the markers PD-1 and PD-L1. Scale bars: 100 μm. (n = 5 per group) **(E)** The mice were administered i.p. injections of IgG (q3d), 50 mg/kg/d TMP195 with or without 200 μg of PD-1 blockade (q3d) or 200 μg PD-1 blockade alone (q3d). Representative photographs of tumors in each group are shown. **(F)** Tumor weight and **(G)** volume were measured in the control group, TMP195 group, PD-1 blockade group and PD-1 blockade plus TMP195 group. Statistical analysis was performed by unpaired Student's t-test. (n = 10 per group) (*P* < 0.05 is statistically significant, *P < 0.05, **P < 0.01, ***P < 0.001)

At present, it is widely accepted that the antitumor mechanism of PD-1/PD-L1 blockade immunotherapy involves the rejuvenation of T cells, but recent studies have demonstrated that there is a complicated relationship between macrophages and the PD-1/PD-L1 pathway during the progression of cancer. Studies have shown that PD-1 expression on macrophages increases with time and with disease progression, and PD-L1 is widely expressed on different cells, including tumor cells, T cells, B cells, macrophages and dendritic cells. In addition, macrophages have crucial effects on the efficacy of PD-1/PD-L1 blocking agents 9, 25. In our study, BMDMs that were induced by LPS and TMP195 showed higher mRNA expression and protein levels of PD-L1 and PD-1 than BMDMs stimulated with LPS only (Figure 7A-B). These results were verified in MC38-transplanted tumor tissue via IHC analysis. The tumors of mice that were treated with TMP195 exhibited higher expression of PD-1 and PD-L1 than those in the DMSO control group (Figure 7C-D). It would be worth studying whether the combination of PD-1 antibody and TMP195 could enhance the efficacy of PD-1 antibody. In the MC38-transplanted tumor model, mice received intraperitoneal injections of IgG, 50 mg/kg TMP195 alone or in combination with 200 μg of the PD-1 blockade, or PD-1 blockade alone. The PD-1 blockade (200 μg) was administered by intraperitoneal injection to each mouse every three days. The first administration of the PD-1 antibody was on the fifth day after the inoculation of MC38 cells and was repeated every 3 days. The administration of TMP195 was the same as described above. On Day 20, the mice were sacrificed, and the tumors were isolated. The tumor weight in the PD-1 blockade combined with TMP195 group was lower than that in the PD-1 antibody group and TMP195 group. The tumor sizes of mice treated with PD-1 blockade and TMP195 were decreased compared with those in the PD-1 blockade group and TMP195 group (unpaired Student's t test) (Figure 7E-G). It indicated that TMP195 could enhance the efficacy of PD-1 blockade on colon cancer.

### PD-1 blockade combined with TMP195 increases M1 macrophage and T lymphocyte infiltration

**Figure 8:**
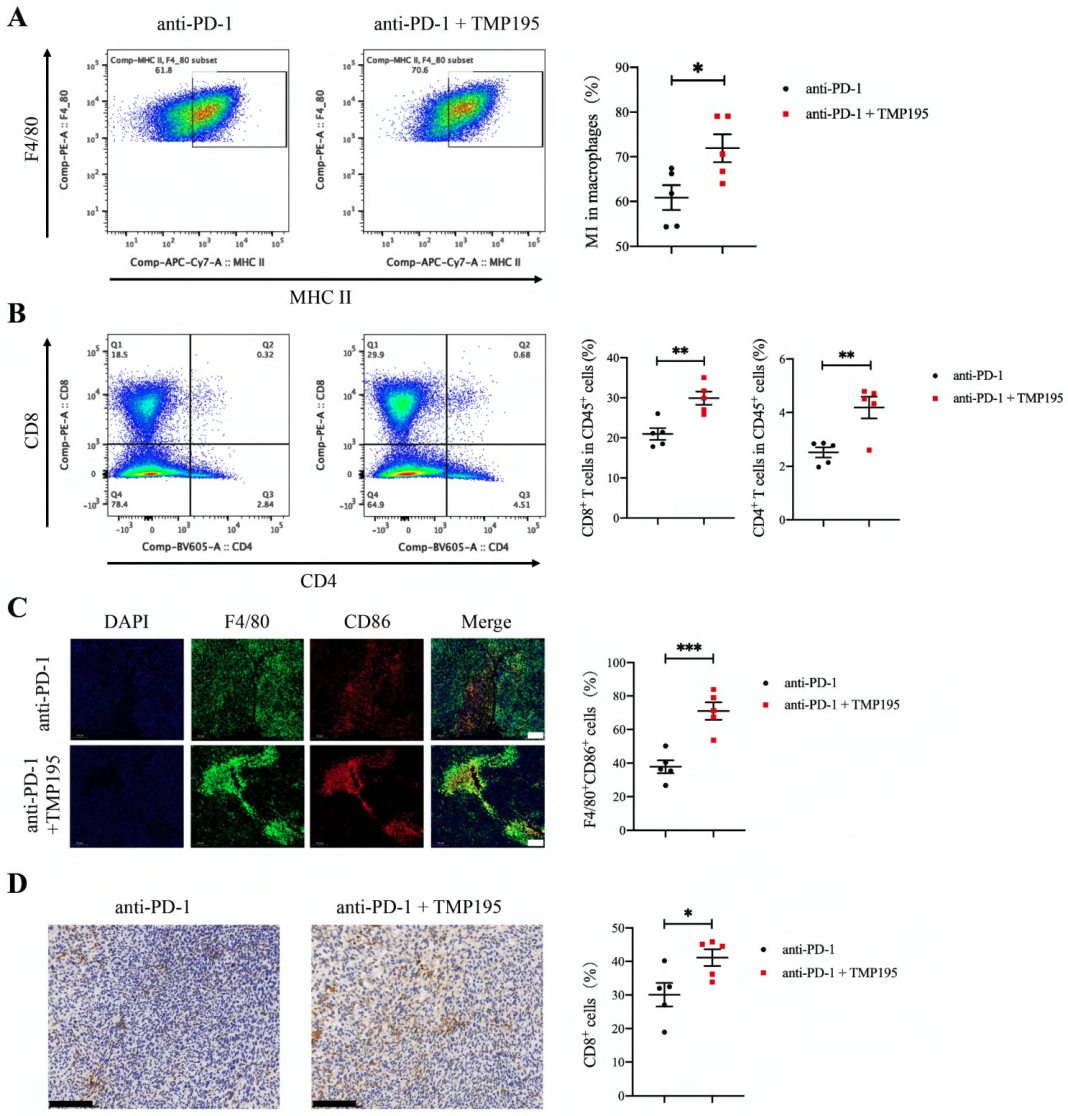
** PD-1 blockade combined with TMP195 increases M1 macrophage and T lymphocyte infiltration.** The mice were inoculated with MC38 cells and treated with PD-1 blockade alone or in combination with TMP195. **(A-B)** Flow cytometry was performed to detect the infiltration of M1 macrophage (CD45^+^CD11b^+^F4/80^+^MHC-II^+^) and T lymphocyte (CD45^+^CD8^+^, CD45^+^CD4^+^). (n = 5 per group) **(C)** Immunofluorescence analysis was performed to identify M1 macrophages with CD86 and F4/80 colocalization in the tumor sections. The proportion of F4/80^+^CD86^+^ cells was plotted as a percentage of F4/80^+^ cells. Representative photographs are shown. Scale bars: 100 μm. (n = 5 per group) **(D)** Cytotoxic T lymphocytes were labeled with CD8 by IHC. Representative photographs are shown. Scale bars: 100 μm. (n = 5 per group) (P < 0.05 is statistically significant, *P < 0.05, **P < 0.01, ***P < 0.001)

As mentioned previously, we found that TMP195 increased the infiltration of M1 macrophages in tumor tissue. The combination of PD-1 blockade and TMP195 had a stronger therapeutic effect on colorectal cancer than monotherapy. Therefore, we hypothesized that TMP195 could increase the infiltration of M1 macrophages and enhance their effects, including activating the tumoricidal function of cytotoxic T lymphocytes. The tumor tissues in this experiment (Figure 7E) were used to further study the infiltration of M1 macrophages and T lymphocytes. The proportion of CD45^+^F4/80^+^CD86^+^ M1 macrophage was analyzed by flow cytometry, and M1 macrophages showed relatively more infiltration in the PD-1 blockade and TMP195 combination group than in the PD-1 blockade group (60.86 ± 2.78 vs. 71.90 ± 3.12%, P < 0.05, Figure 8A). The infiltration of T lymphocytes was also analyzed. The proportion of both CD8^+^ cytotoxic T cells and CD4^+^ helper T cells was increased in the PD-1 blockade plus TMP195 group compared with that in the PD-1 blockade group (20.96 ± 1.44 vs. 29.88 ± 1.65%, P < 0.01; 2.52 ± 0.19 vs. 4.19 ± 0.41%, P < 0.01, Figure 8B). In the tumor sections stained with immunofluorescence, M1 macrophages showed relatively more infiltration in the PD-1 blockade plus TMP195 group than in the PD-1 blockade group (37.82 ± 3.82 vs. 71.01 ± 5.21%, P<0.001, Figure 8C). Furthermore, increased numbers of cytotoxic T lymphocytes labeled with CD8 were recruited and activated in the PD-1 blockade plus TMP195 group (30.12 ± 3.50 vs. 41.10 ± 2.52%, P<0.05, Figure 8D). These results indicated that the role of TMP195 in promoting PD-1 blockade efficacy might be due to the promotion of M1 macrophages polarization and T lymphocyte infiltration.

## Discussion

Histone deacetylases are overexpressed in several types of cancer, such as colorectal cancer, breast cancer and lung cancer. Previous work has reported that a variety of HDAC inhibitors have therapeutic effects on several types of cancer, and vorinostat and romidepsin have been approved by the FDA. The novel IIa HDAC inhibitor TMP195 has been shown to be effective in the treatment of breast cancer, mainly through a new strategy that induces the recruitment and differentiation of highly phagocytic and stimulatory macrophages within tumors 21. Although vorinostat and romidepsin, which are class I HDAC inhibitors, have achieved great success in the clinic, there are still some disadvantages, such as poor pharmacokinetics and selectivity 20. Compared with class I HDAC inhibitors and pan-HDAC inhibitors, classic IIa HDAC selective inhibitors such as MC1568 and MC1575 have improved these disadvantages to some extent and reduced the cytotoxic effects of HDACis on cells. Even so, MC1568 and MC1575 have been reported to inhibit the proliferation of melanoma cells and in ER^+^ breast cancer cells 26, 27. As a novel class IIa HDACi, TMP195 exhibits stronger selectivity with fewer off-targets and no overt cytotoxic effects than other treatments because the hydroxamic Zn^2+^ binding domain in classic IIa HDACis replaced by a trifluoromethyloxadiazolyl group (TFMO) 27. However, the role of TMP195 in colorectal cancer remains unclear.

In this study, the therapeutic effect of TMP195 on colorectal cancer was significant in two colorectal mouse models, including a CAC model and an MC38 subcutaneous graft tumor model. Mechanistically, TMP195 had no significant direct effect on proliferation or apoptosis in colon cancer cell lines, which suggests that the therapeutic effect of TMP195 on colorectal cancer may not be directly related to the tumor cells. In various tumors, the HDAC inhibitor trichostatin-A has been reported to reshape the tumor immune microenvironment, decrease the infiltration of MDSCs and the suppressive activity of TAM 28. Through TIMER 2.0 database mining, there was a common and significant correlation between class IIa HDAC genes (HDAC4, -5, -7, and -9) and macrophages, especially M1 macrophages, in COAD and READ (Supplemental Data Figure 2A-F). Therefore, this study analyzed the difference in the tumor immune microenvironment between the TMP195-treated group and the control group to examine the mechanism of TMP195. In this study, the results showed that TMP195 could decrease the proportion of macrophage-to-leukocyte in the tumors and the surrounding lamina propria in CAC mice, but no significant change in the proportions of T lymphocytes were observed. When macrophages were partly depleted by clodronate liposomes, TMP195 lost its therapeutic effect on colorectal cancer. Further study of the immune microenvironment in MC38 colon carcinoma-transplanted tumors and CAC tumors showed that the proportion of M1 macrophages relative to total macrophages and the secretion of M1 macrophage-associated inflammatory cytokines was increased. It is known that macrophages play a complex role in tumorigenesis and tumor progression, and M1-type macrophages mainly exert antitumor effects and can release inflammatory cytokines 29. We speculate that the proportion change of macrophages may be due to the reprogramming of macrophage polarization, and the polarization reshape of macrophages may play an important role in the efficacy of TMP195 on colorectal cancer. This study not only investigated the regulation of macrophages by TMP195 in tumor microenvironment, but also examined its effects on the function of M1 macrophages *in vitro*. TMP195 enhanced BMDMs M1 polarization induced by LPS and increased the release of inflammatory cytokines. In addition, we evaluated the activation of M1 macrophage-associated cytokines signaling pathways, such as the NF-κB and p38/JNK MAPK signaling pathways. The results showed that TMP195 could promote the phosphorylation of NF-κB, p38 and JNK. Based on these results, we hypothesize that TMP195 plays an important role in the treatment of colorectal cancer by promoting the polarization of macrophages to the antitumor phenotype and the secretion of inflammatory cytokines.

Previous studies have reported that immunotherapy is effective in B-cell lymphoma, but many patients have shown resistance to PD-1 blockade. The combination of PD-1 blockade and HDACi improved the efficacy of PD-1 blockade in patients with PD-1 blockade resistance. Histone deacetylase inhibition can sensitize resistant B-cell lymphomas patients to PD-1 blockade 30, 31. In solid tumors, such as breast cancer, the addition of TMP195 to the PD-1 blockade regimen yielded a significant reduction in tumor burden compared to PD-1 blockade alone 21. In NSCLC patients that were treated with PD-1 blockade therapy, high expression of PD-L1 in macrophages was associated with longer overall survival 32. Inspired by previous studies, our study showed that TMP195 could increase the expression of PD-1 and PD-L1 in BMDMs and tumor tissues, suggesting the possible effectiveness of the ICI and TMP195 combination in colorectal cancer. In the subcutaneous transplanted tumor model, tumors from mice treated with TMP195 and PD-1 blockade were significantly smaller than those from mice treated with TMP195 or PD-1 blockade alone.

According to our findings, the HDAC inhibitor TMP195 is likely to be a new choice for the treatment of colorectal cancer. In the treatment of colorectal cancer, TMP195 can increase the efficacy of PD-1 blockade, suggesting that the combination of TMP195 and immunotherapy provides a potential option for colorectal cancer treatment.

## Supplementary Material

Supplementary methods and figures.

## Figures and Tables

**Table 1:** Primer sequences for RT-PCR.

Genes	Sequences
IL-12	F: GGAAGCACGGCAGCAGAATA R: AACTTGAGGGAGAAGTAGGAATGG
TNF-α	F: TACTGAACTTCGGGGTGATCGGTCC R: CAGCCTTGTCCCTTGAAGAGAACC
iNOS	F: CAGCTGGGCTGTACAAACCTT R: CATTGGAAGTGAAGCGTTTCG
PD-1	F: ACCCTGGTCATTCACTTGGG R: CATTTGCTCCCTCTGACACTG
PD-L1	F: GCTCCAAAGGACTTGTACGTG R: TGATCTGAAGGGCAGCATTTC
β-Actin	F: GCTGACCTGATGGAGTTGGA R: GCTACTTGCTCTTGCGTGAA

**Table 2:** Antibodies used in this study.

Antibody	Supplier	Cat.No.	Dilution	Application
p38 MAPK	CST	8690	1:1000	WB
Phospho-p38 MAPK	CST	4511	1:1000	WB
JNK2	CST	9258	1:1000	WB
P-JNK	CST	4668	1:1000	WB
NF-κB p65	CST	8242	1:1000	WB
Phospho-NF-κB p65	CST	3033	1:1000	WB
β-actin	CST	8457	1:1000	WB
PD-L1	CST	13684	1:1000	WB/IHC
PD-1	CST	84651	1:1000	WB/IHC
Histone H3	CST	4499	1:1000	WB
Acetyl-Histone H3	CST	7627	1:1000	WB
F4/80	CST	70076	1:400	IHC
CD8	CST	85336	1:400	IHC

## Abbreviations

CRC: colorectal cancer
CAC: colitis-associated colorectal cancer
HDAC: histone deacetylase
HDACis: histone deacetylase inhibitors
TAMs: tumor-associated macrophages
PD-1: programmed cell death protein 1
PD-L1: programmed cell death protein 1 ligand 1
DSS: dextran sulfate sodium salt
AOM: azoxymethane
DMSO: dimethyl sulfoxide
IHC: immunohistochemistry
IF: immunofluorescence
iNOS: inducible nitric oxide Synthase
TNFα: tumor necrosis factor α
IL-12: interleukin-12
IL-6: interleukin-6
IL-1β: interleukin-1β
BMDMs: Bone marrow-derived macrophages
mRNA: messenger RNA
FBS: fetal bovine serum
PBS: phosphate-buffered saline
