# Supplementary material for: TMP195 Exerts Antitumor Effects on Colorectal Cancer by Promoting M1 Macrophages Polarization
Source: Int J Biol Sci. 2022 Sep 6;18(15):5653–66. doi: 10.7150/ijbs.73264 (PMC9576521; doi:10.7150/ijbs.73264)
Supplement: Supplementary file 1 — Supplementary methods and figures. [file ijbsv18p5653s1.pdf]

Supplemental material

Cell proliferation and apoptosis assays

Cells were seeded into 96-well plates in medium containing 10% FBS at a density of 4000 cells/well, and the cells were stimulated with TMP195 24 hours later and then analyzed with a Cell Counting Kit-8 assay (C0040, Beyotime Biotech, Shanghai, China) every 24 hours. Ten microliters of CCK8 assay reagent were added to each well, and the plate was incubated at 37 ˚C for 1 hour. The results were quantified by spectrophotometry at a wavelength of 450 nm.

To determine cell apoptosis, cells were seeded in 6-well plates at a density of 4×10^5/well and stimulated with different concentrations of TMP195 for 24 hours. The cells were stained with an Apoptosis Kit (annexin V-FITC and PI) (Lianke Biotech, China) and analyzed by flow cytometry.

Soft agar colony formation assay

The 1.25% and 0.825% agar solutions were prepared, sterilized at 121 ℃ for 20 minutes, and then kept in a water bath at 42 ℃. Then, 0.5% agar containing DMEM and FBS was added to each well in a 12-well plate and solidified at 4 ℃. A total of 1×10^5 cells/well were mixed with 500 μl of 0.33% agar containing DMEM and FBS and added to the 12-well plate. After the agar was solidified, culture medium (DMEM) containing 10% FBS and TMP195 was added to each well. After colonies with appropriate sizes were formed, an inverted microscope was used for imaging.

Immune infiltration database analysis

The association between immune infiltration and gene expression was determined using the TMIER 2.0 database, which is a high-quality and public database for the systematic analysis of immune infiltration across various cancer types. Systematic analysis of immune infiltration in colon adenocarcinoma (COAD) and rectal adenocarcinoma (READ) TCGA samples was demonstrated in this study.

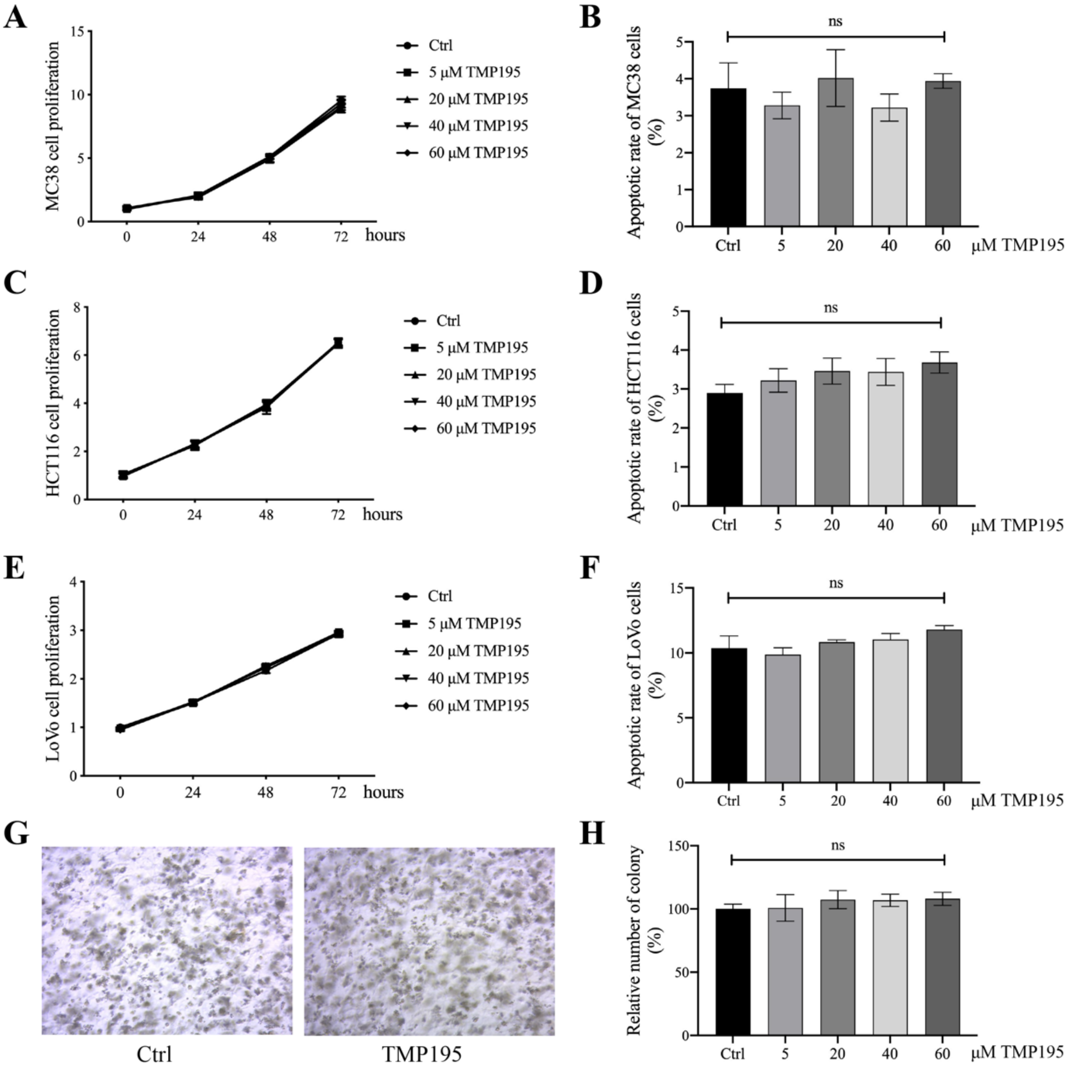

Supplemental Data Figure 1. TMP195 has no direct effect on colorectal cancer cell proliferation or apoptosis in vitro. MC38, HCT116 and LoVo cells were cultured in medium containing 5, 20, 40, or 60 μM TMP195 or DMSO. **(A, C, E)** The cell-counting-kit 8 (CCK8) assay was used to examine the proliferation of MC38, HCT116 and LoVo cells. Six replicates were set for each experimental group. **(B, D, F)** The apoptotic rates of MC38, HCT116 and LoVo cells were examined by PI staining and flow cytometry. Five replicates were set for each experimental group. **(G-H)** A soft agar colony formation assay was performed. The relative colony formation rate of MC38 cells was evaluated. ($P < 0.05$ is statistically significant)

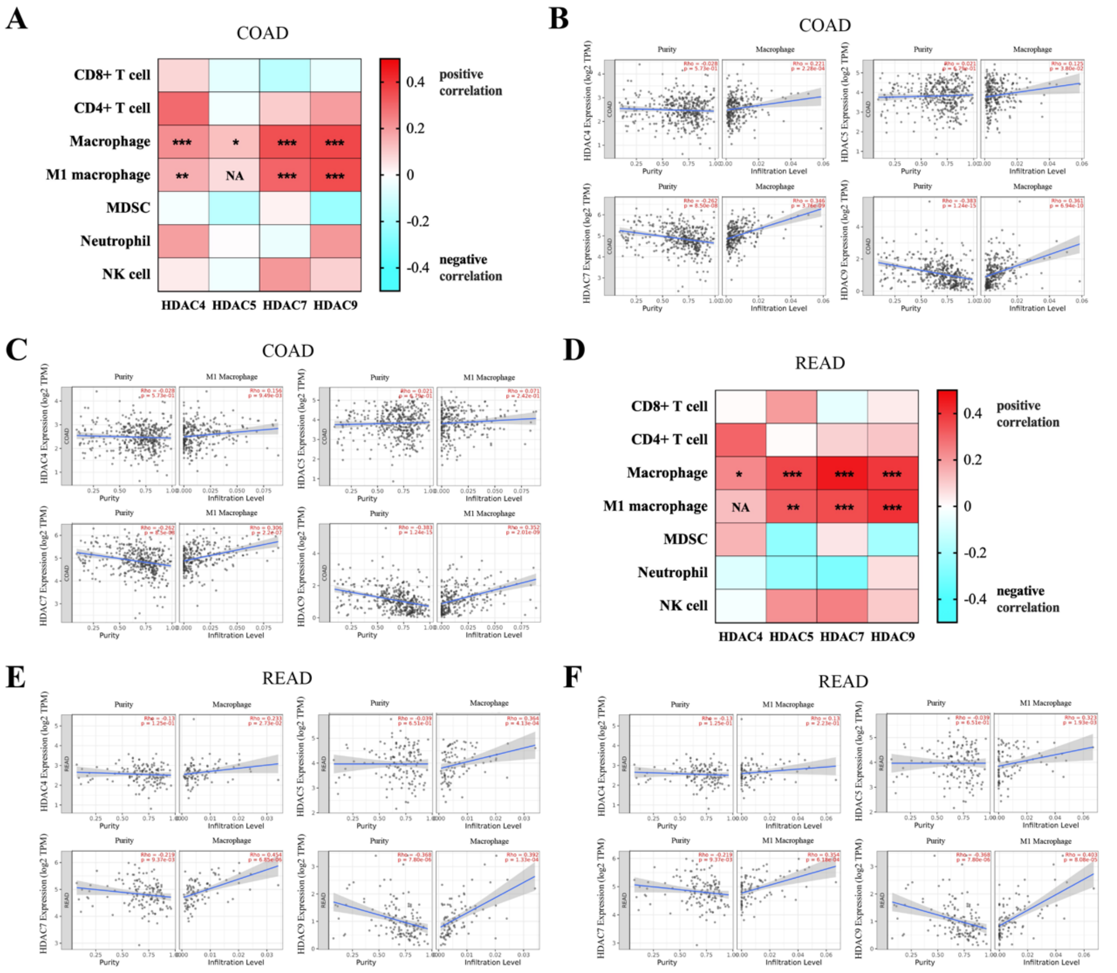

Supplemental Data Figure 2. Correlation between immune infiltration and class IIa HDAC in COAD and READ. **(A)** Correlations between immune cells including cytotoxic T cells, helper T cells, macrophages, M1 macrophages, MDSCs, neutrophils and NK cells, and the genes encoding HDAC4, HDAC5, HDAC7, and HDAC9 in COAD were analyzed with TIMER 2.0 system. Correlations between **(B)** macrophages/ **(C)** M1 macrophages and HDAC4, HDAC5, HDAC7, and HDAC9 are showed in detail in COAD. **(D)** Correlations between immune cells including $CD8^{+}$ T cells, $CD4^{+}$ T cells, macrophages, M1 macrophages, MDSCs, neutrophils and NK cells and the genes encoding HDAC4, HDAC5, HDAC7, and HDAC9 in READ were analyzed with the TIMER 2.0 system. Correlations between **(E)** macrophages/ **(F)** M1 macrophages and HDAC4, HDAC5, HDAC7, and HDAC 9 are shown in detail in READ. ($P < 0.05$ is statistically significant, $*P < 0.05$, $**P < 0.01$, $***P < 0.001$)
